# Supplementary material for: Reconciling Mining with the Conservation of Cave Biodiversity: A Quantitative Baseline to Help Establish Conservation Priorities
Source: PLoS One. 2016 Dec 20;11(12):e0168348. doi: 10.1371/journal.pone.0168348 (PMC5173368; doi:10.1371/journal.pone.0168348)
Supplement: S1 Dataset — (ZIP) [file pone.0168348.s002.zip › Taxa/Serra Norte/SN_2013/N5S/Lista N5S-20.pdf]

CAVIDADE N5S-0020

Classe	Ordem	Fam/Outros	Gên/Outros	Espécie	S-U	U-S	Zon.
Arachnida	Acari	Eupodidae		sp.1	X		E
Arachnida	Amblypygi	Phrynidae	*Heterophrynus*	*longicornis*	4	X	P
Arachnida	Araneae	Caponiidae		sp.1	X	X	P
Arachnida	Araneae	Corinnidae		jovem	1		P
Arachnida	Araneae	Ochyroceratidae	*Ochyrocera*	sp.1	X	X	P/A
Arachnida	Araneae	Ochyroceratidae	*Speocera*	sp.1	X		P/A
Arachnida	Araneae	Salticidae		sp.2	X		E
Arachnida	Araneae	Theraphosidae		jovem	1		P
Arachnida	Araneae	Theridiosomatidae	*Plato*	sp.1	X	X	P
Arachnida	Araneae	Uloboridae		jovem	X		E
Arachnida	Opiliones	Escadabiidae		sp.2	X	X	P/A
Arachnida	Pseudoscorpiones	Bochicidae		sp.		X	
Arachnida	Pseudoscorpiones	Chernetidae		sp.	X	X	
Arachnida	Pseudoscorpiones	Chthoniidae	*Pseudochthonius*	sp.2		X	
Chilopoda	Scolopendromorpha	Scolopocryptopidae	*Dinocryptops*	*miersii*		X	
Chilopoda	Scolopendromorpha	Scolopocryptopidae	*Tidops*	sp.1	1		E
Chilopoda	Scolopendromorpha	Scolopocryptopidae		sp.1	1		E
Chilopoda	Scutigeromorpha	Pselliodidae	*Sphendononema*	jovem	2		P
Diplopoda	Polydesmida	Pyrgodesmidae		sp.1	X	X	P/A
Diplopoda	Spirostreptida	Pseudonannolenidae	*Pseudonannolene*	sp.1		X	
Entognatha	Collembola	Paronellidae		sp.4	X		P/A
Insecta	Blattodea			jovem	4		E/P
Insecta	Coleoptera	Staphylinidae	Pselaphinae	sp.12	X		P
Insecta	Coleoptera	Staphylinidae		sp.28	X		E
Insecta	Diptera	Culicidae	*Culex*	sp.	X		P
Insecta	Diptera	Dolichopodidae		sp.	X		P
Insecta	Diptera	Psychodidae	Phlebotominae	sp.	X		P
Insecta	Heteroptera	Ochteridae		jovem	X		P
Insecta	Heteroptera	Reduviidae	Emesinae	sp.1	1		P
Insecta	Homoptera	Cixiidae		sp.5	X		P
Insecta	Hymenoptera	Braconidae		sp.1	X		E
Insecta	Hymenoptera	Formicidae	*Gnamptogenys*	sp.1	X		E
Insecta	Hymenoptera	Formicidae	*Pachycondyla*	sp.1	X		P
Insecta	Hymenoptera	Formicidae	*Pachycondyla*	sp.2	X		P
Insecta	Hymenoptera	Formicidae	*Proceratium*	sp.1		X	
Insecta	Hymenoptera	Formicidae	*Solenopsis*	sp.		X	
Insecta	Isoptera	Rhinotermitidae	*Rhinotermes*	sp.1	X		P
Insecta	Isoptera	Termitidae	*Nasutitermes*	sp.	X		E
Insecta	Lepidoptera	Tineoidea		sp.1	X	X	P
Insecta	Neuroptera	Myrmeleontidae		jovem	X		E
Insecta	Orthoptera	Phalangopsidae	*Paraclodes*	sp.	2		E
Insecta	Orthoptera	Phalangopsidae	*Phalangopsis*	sp.	15	X	
Insecta	Psocoptera	Epipsocidae	*Epipsocus*	sp.2	X		P
Gastropoda	Pulmonata	Subulinidae	*Lamellaxis*	sp.		X	
Malacostraca	Isopoda	Scleropactidae	*Circoniscus*	*buckupi*	X		P
Malacostraca	Isopoda	Scleropactidae		sp.		X	
Symphyla		Scutigerellidae		sp.	X		P
Amphibia	Anura	Strabomantidae	*Pristimantis*	cf. *fenestratus*	2		E/P
Mammalia	Chiroptera	Emballonuridae	*Peropteryx*	*kappleri*	4		P/A
